# Saponins of Korean Red Ginseng May Protect Human Skin from Adipokine-Associated Inflammation and Pigmentation Resulting from Particulate Matter Exposure

**DOI:** 10.3390/nu14040845

**Published:** 2022-02-17

**Authors:** Ik Jun Moon, WooHyeong Kim, Su Yeon Kim, JeongHyeon Lee, Hanju Yoo, Seunghyun Bang, Youngsup Song, Sung Eun Chang

**Affiliations:** 1 Department of Dermatology, Asan Medical Center, University of Ulsan College of Medicine, Seoul 05505, Korea; ikjun.moon@gmail.com (I.J.M.); kwh3114@naver.com (W.K.); u2u2star@naver.com (S.Y.K.); zoo535353@gmail.com (J.L.); julia_yoo@hanmail.net (H.Y.); bangsh1037@gmail.com (S.B.); 2 Bio-Medical Institute of Technology (BMIT), University of Ulsan College of Medicine, Seoul 05505, Korea; 3 Department of Biomedical Sciences, Asan Medical Center, University of Ulsan College of Medicine, Seoul 05505, Korea

**Keywords:** particulate matter, leptin, melanogenesis, inflammation, *Panax ginseng*, saponins

## Abstract

Background: Exposure to airborne particulate matter (PM) is an ever-increasing concern worldwide. Strategies to counter the detrimental effects that follow cutaneous exposure to PM, such as induction of pigmentation, inflammation, and alterations in adipokine profile, need to be investigated further. Korean red ginseng (KRG) extracts and individual ingredients have been demonstrated to play an effective role in suppression of ROS, inflammation, and resultant skin aging. In addition, recent investigations revealed that Rg3 and Rf saponins work as antimelanogenic agents. In this study, we investigated whether saponins of KRG can protect against or reverse the PM-induced detrimental effects. Methods: The biological effects of PM and saponins were evaluated both in vitro and ex vivo. Cell viability and intracellular ROS levels were determined in normal human epidermal melanocytes (NHMs), human epidermal keratinocytes (NHKs), and their cocultures. Experiments to demonstrate the protective properties of saponins against consequences of exposure to PM were performed. Melanin assay, quantitative real-time PCR, and Western blotting were carried out to determine the effects on melanogenesis and the implicated molecular signaling pathways. Results: Exposure to PM resulted in decreased keratinocyte viability, which was coupled with augmented oxidative stress. These changes were attenuated by treatment with saponins. PM exposure resulted in increased expression of leptin, which was reduced by saponins. Moreover, PM exposure led to increased melanin production in a coculture model, which was mitigated by treatment with saponins. Treatment with saponins resulted in a decrease in matrix metalloproteinase (MMP) levels after exposure to PM. Conclusion: Saponins of KRG can protect the skin from the harmful effects of PM exposure by reducing levels of ROS, leptin, inflammatory cytokines, and melanin.

## 1. Introduction

Growing public awareness of the harmful effects of particulate matter (PM) exposure on various organs and systems has led to active research to determine the mechanisms of damage and prevention or treatment, with special focus on the respiratory system. It has been clinically and experimentally demonstrated that because the respiratory system is inevitably exposed to airborne pollutants such as PM, underlying respiratory diseases such as asthma and chronic obstructive pulmonary diseases and lung cancer are exacerbated by PM exposure [1,2]. Another organ that is at high risk of negative effects due to PM exposure is the eye. In fact, eye diseases such as keratoconjunctivitis have been shown to be induced by contact with PM [3]. The skin is also in direct contact with the environment, and atmospheric PM is thus thought to be capable of damaging the skin. The harmful health impact of PM exposure is not confined to those organs exposed to PM. In fact, PM exposure has been associated with several systemic conditions, including dyslipidemia, obesity, insulin resistance, and diabetes mellitus [4,5,6]. Among various mechanisms that link exposure to PM to these metabolic disorders, changes in adipokine levels, including adiponectin and leptin, have been highlighted in previous studies [7,8].

In the past decade, clinical reports have shown a correlation between atmospheric PM concentrations and the progression of extrinsic skin aging. In previous studies, signs of skin aging as assessed by both pigmentary change and decreased skin laxity were associated with PM exposure [9,10]. However, the biological mechanistic link between PM and pigmentation and/or extrinsic skin aging remains largely unclear. Most studies suggest that the link is reactive oxygen species (ROS). It has been demonstrated that PM increases oxidative stress in skin cells and induces ROS-dependent inflammation [11]. Another study suggested a role of PM-induced autophagy in skin aging [12].

In addition to searching for a mechanistic link, there is an urgent need to develop strategies to prevent or reverse detrimental effects such as the induction of adipokine-associated inflammation and pigmentation caused by cutaneous exposure to PM. In particular, anti-PM and antimelanogenic strategies need to be actively explored since people with dark skin types in Korea and other East Asian countries are at high risk of exposure to PM produced domestically and PM coming from neighboring countries. Among several mechanisms implicated in excessive cutaneous pigmentation in Asian skin, the important roles of endothelin and adipokines have been reported previously [13,14,15].

Among various naturally derived products, Korean red ginseng (KRG) extracts and individual ingredients have been demonstrated to play a role in the suppression of ROS, inflammation, and resultant skin aging. *Panax ginseng*, commonly known as Korean red ginseng, has served as a key ingredient in Asian herbal medicine for more than 2000 years. Owing to its popularity, researchers have aimed to identify the active substances related to the beneficial effects of KRG. The main active substances of KRG can be divided into two categories: saponin fraction and non-saponin fraction. The saponins in KRG are a heterogeneous group of compounds including ginsenosides, which are known as the pivotal substances of ginseng extracts. To date, more than 40 ginsenosides have been isolated and identified among the saponins. Ginsenosides have been investigated for their antioxidant, anti-inflammatory, antineoplastic, and even hair-growth-promoting effects in dermatological studies [16,17,18,19,20]. In addition, some saponin (ginsenoside) ingredients and nonsaponin ingredients have been reported to affect skin melanogenesis in either antimelanogenic or promelanogenic ways for the treatment of hyper- or hypopigmentation disorders. In particular, Rg3 and Rf ginsenosides were revealed to work as antimelanogenic agents.

In order to find effective oral or topical agents for the protection of human skin from airborne-PM-induced inflammation and pigmentation, we hypothesized that saponins of KRG may protect or reverse the negative impacts of exposure to PM, considering that KRG is known to reduce both ROS and inflammation, which are the main pathogeneses of PM-induced skin disorders [11]. Taking a simulation-based approach to the investigation of real-world PM-induced skin disorders, this study was designed to investigate the effect of exposure to three types of commercially available samples of PM measuring between 2.5 μm and 10 μm in size (PM10) and one type of local PM measuring less than 10 μm from outside our research institution. Furthermore, as the skin is a paracrine organ and interacts biologically with other cells in responses to PM, skin constituent cells including human keratinocytes and melanocytes, keratinocyte-conditioned media-treated dermal fibroblasts, cocultures of keratinocytes and melanocytes, and ex vivo skin cultures were used in this study.

## 2. Materials and Methods

### 2.1. Materials

Both saponins and nonsaponins of Korean red ginseng (KRG) were provided by the Korea Ginseng Corporation (Daejeon, Korea). The antibodies for tyrosinase, microphthalmia-associated transcription factor (MITF), and 6-carboxy-2′,7′-dichlorodihydrofluorescein diacetate (CM-H2DCFDA) were purchased from Thermo Fisher Scientific (Invitrogen, Waltham, MA, USA). The antibody for MMP-1 was obtained from GeneTex (Irvine, CA, USA). H_2_O_2_, lipopolysaccharide (LPS), and the antibodies for beta-actin and *N*-acetyl-l-cysteine were obtained from Sigma-Aldrich Co. (St. Louis, MO, USA). Dulbecco’s modified Eagle’s medium (DMEM) and fetal bovine serum (FBS) were purchased from WelGENE Inc. (Gyeongsan, Korea).

### 2.2. Particulate Matter Collection

We collected and selected local samples of particulate matter (PM) (referred to as local PM10 in this manuscript) measuring less than 10 μm outside the Asan Research Institution building, located in an urban area in Seoul, Korea, from January 2019 to March 2019, with reference to the previous publication of Chung et al. [21]. The collection site was 200 m away from a two-way street with total of eight lanes. Han River, which is more than 1 kilometer wide, is located 500 m away from the collection site. In addition, three commercially available types of PM measuring between 2.5 μm and 10 μm in size (PM10) were purchased and tested to determine which PM increased melanogenesis to the greatest degree in compared to local PM. Urban aerosols (CRM No. 28) were purchased from the National Institute for Environmental Studies (NIES), Ibaraki, Japan. Certified and reference values for the elements and polycyclic aromatic hydrocarbons (PAHs) constituting the material are available online on the NIES webpage (https://www.nies.go.jp/labo/crm-e/index.html, accessed on 7 February 2022). The standard reference materials 1648a (pmA) and 1649b (pmB) were purchased from the National Institute of Standards and Technology (NIST, Gaithersburg, MD, USA). These both consist primarily of PAHs and heavy metals, and their constitutions are available on the NIST webpage (http://www.nist.gov/srm, accessed on 7 February 2022).

### 2.3. Cell Culture and Coculture

Normal human epidermal melanocytes (NHMs; neonatal, moderately pigmented) were cultured in Medium 254 supplemented with human melanocyte growth supplement (HMGS; Cascade Biologics, Invitrogen) at 37 °C and 5% CO_2_. NHMs were used between passages 3 and 5. Normal human epidermal keratinocytes (NHKs; neonatal) were cultured in Epilife medium supplemented with human keratinocyte growth supplement (HKGS; Cascade Biologics, Invitrogen) at 37 °C and 5% CO_2_. NHKs were used between passages 3 and 5. Cells from the murine macrophage line RAW264.7 were maintained in Dulbecco’s modified Eagle’s medium (DMEM) supplemented with 10% FBS and 1% antibiotic–antimycotic at 37 °C in 5% CO_2_. A coculture of human keratinocytes and melanocytes was generated in keratinocyte medium at a seeding ratio of 1:1 (for melanin assay, Western blotting, and intracellular signaling assay). NHMs were seeded onto a 6-well plate at a density of 6 × 10^4^ or 3 × 10^5^ cells per well. On the next day, NHKs were added to each well at a density of 3 × 10^5^ cells for the coculture. After 24 h, PM was added and melanin content was measured after 3 or 5 days.

### 2.4. Cell Viability Assay

Cell viability was measured using MTT assays. After starvation for 24 h, cells were treated with PM for 24 h. MTT solution (2.5 mg/mL) was added to the culture medium and incubated for 4 h. The resulting formazan crystals were dissolved in DMSO. Absorbance was measured using a microplate reader (Molecular Devices, Sunnyvale, CA, USA) at 570 nm.

### 2.5. Measurement of Melanin Contents

Cells were dissolved in 1 N NaOH at 100 °C for 30 min and centrifuged at 13,000 rpm for 1 min. The optical densities (OD) of the supernatants were measured at an absorbance of 405 nm using a microplate reader. The protein content of the samples was determined using the Bradford assay (Bio-Rad, Hercules, CA, USA). Melanin content was calculated by normalizing with the protein concentrations.

### 2.6. Intracellular ROS Assay

Intracellular ROS levels were measured using the DCF-DA assay. NHKs were seeded onto a 96-well plate at 1.8 × 10^4^ cells per well and were treated with 100 μg/mL PM10 and 50 μg/mL saponins or 50 μg/mL nonsaponins for 24 h. After 24 h, the medium was removed and cells were washed twice with PBS. Cells were then incubated with 10 μM DCF-DA for 30 min at 37 °C in the dark and washed twice with PBS. Fluorescence intensity was then measured at 485 nm excitation and at 535 nm emission using a microplate reader (VICTOR Multilabel Plate Reader, PerkinElmer, Waltham, MA, USA). For short-time ROS assays and to examine the reversal reduction of ROS by the known antioxidant *N*-acetyl-l-cysteine, NHEKs were seeded onto a 96-well plate at 3 × 10^4^ cells per well and were pretreated with 50 μg/mL saponin, 50 μg/mL nonsaponin, or 1 mM *N*-acetyl-l-cysteine for 1 h and then treated with 100 μg/mL PM10 for 3 h. For comparison of the effect between pretreatment and simultaneous treatment, the cells were simultaneously treated with 50 μg/mL saponin, 50 μg/mL nonsaponin, or 1 m *N*-acetyl-l-cysteine and 100 μg/mL PM10 for 3 h. After the indicated time, the medium was removed and cells were washed twice with PBS. Cells were then incubated with 10 μM DCF-DA for 30 min at 37 °C in dark and washed twice with PBS. Fluorescence intensity was then measured at 485 nm excitation and at 535 nm emission using a microplate reader (VICTOR Multilabel Plate Reader, PerkinElmer, Waltham, MA, USA).

### 2.7. Quantitative Real-Time Polymerase Chain Reaction (PCR)

Total cellular RNA was extracted from cells using FavorPrep^TM^ Total RNA Purification Mini Kit according to the manufacturer’s instructions (Favorgen, Ping Tung, Taiwan). Following extraction, the quantity and quality of the RNA were determined using a NanoDrop^®^ ND-1000 spectrophotometer (ND-1000, NanoDrop Technologies, Wilmington, DE, USA). Single-stranded cDNA was synthesized from 1 μg of total RNA using a Revert Aid First Strand cDNA Synthesis Kit according to the manufacturer’s instructions (Thermo Scientific, Rockford, IL, USA). Quantitative real-time PCR (qRT-PCR) was performed using the LightCycler^®^ 480II system coupled with SYBR Green chemistry (Roche Applied Science, Penzberg, Germany). In terms of qRT-PCR settings, initial denaturation was performed at 95 °C for 5 min, followed by 55 cycles of amplification at 95 °C for 10 s, 60 °C for 10 s, and 72 °C for 10 s. The cDNA was amplified using the following primers:
**Name****Forward (5′ to 3′)****Reverse (5′ to 3′)**Interleukin (IL)-1αAGGGCGTCATTCAGGATGAACGCCAATGACTCAGAGGAAGAIL-1βTCCCCAGCCCTTTTGTTGATTAGAACCAAATGTGGCCGTGIL-6AGACAGCCACTCACCTCTTCAGTTCTGCCAGTGCCTCTTTGCTGIL-8AACCCTCTGCACCCAGTTTTCACTGAGAGTGATTGAGAGTGGACTNF-αAGCTGCCCCTCAGCTTGAGCCCAGGGACCTCTCTCTAATCAMMP-1CTCTGGAGTAATGTCACACCTCTTGTTGGTCCACCTTTCATCTTCMMP-2GATACCCCTTTGACGGTAAGGACCTTCTCCCAAGGTCCATAGCMMP-3CGGTTCCGCCTGTCTCAAGCGCCAAAAGTGCCTGTCTTRPLPOGGCGACCTGGAAGTCCAACTCCATCAGCACCACAGCCTTCSCFTGGTGGCAAATCTTCCAAAAGCAATGACTTGGCAAAACATCCAET-1AAGGCAACAGACCGTGAAAATCGACCTGGTTTGTCTTAGGTGLeptinGCTGTGCCCATCCAAAAAGTCCCCCAGGAATGAAGTCCAAACCGADIPOQACCAGGAAACCACGACTCAACGATGTCTCCCTTAGGACCA

### 2.8. Western Blotting

Cells were lysed in protein lysis buffer (Intron Biotechnology, Seongnam, Korea) and centrifuged at 13,000 rpm for 10 min. Protein concentrations in the supernatant were determined using a bicinchoninic acid protein assay kit. Next, 20 μg of proteins were separated per lane by SDS–polyacrylamide gel electrophoresis and blotted onto nitrocellulose membranes. Blots were incubated with the appropriate primary antibodies at a dilution of 1:1000, and further incubated with horseradish-peroxidase-conjugated secondary antibody. Bound antibodies were detected using an enhanced chemiluminescence kit (Pierce Biotechnology, Rockford, IL, USA). Image analysis was used to determine the relative band densities, using Image J software (https://imagej.nih.gov/ij/, accessed on 7 February 2022).

### 2.9. Ex Vivo Human Skin Explant Model

Discarded full-thickness skin was obtained from four women aged 30–50 years after abdominoplasty at Asan Medical Center. Informed patient consent and approval by the Institutional Review Board were obtained. The whole-skin samples were treated with PM (1648a) or nothing (control). A sterilized stainless steel grid was placed on a 6-well plate and the skin specimens were placed on the stainless steel grid. DMEM supplemented with 10% FBS and 10% antibiotics was used to fill the plate up to the stainless steel grid. After 6 days of culture in an incubator at 37 °C with 5% CO_2_, the specimens were fixed in 10% formalin and embedded in paraffin section. Melanin was detected with Fontana-Masson staining.

### 2.10. Production of Preconditioned Media

Preconditioned medium from the culture of HaCaT and HDF cells was produced according to the method previously described by Fernando et al. [22] To prepare PM10 samples, PM10 was dissolved in DMSO, emulsified, and diluted to 100 μg/mL using PBS. HaCaT cells were seeded at a concentration of 1 × 10^5^ cells/mL and incubated for 24 h. After 1 h, the cells were induced with PM10-containing medium and incubated for half an hour. To remove PM-containing medium, the cells were washed twice with new culture medium and the medium was replaced with new medium. After 24 h of the incubation period, the resulting cell medium was collected, filtered, and stored at −80 °C for future use. In the meantime, HDF cells were seeded at 1 × 10^5^ cells/mL concentration for 24 h in well plates. The preconditioned media from corresponding HaCaT cell treatment groups were applied to the HDF cells and incubated for 30 min. The wells were then emptied and washed twice with new culture medium to remove the remaining preconditioned medium and the medium was replaced with new culture medium. This was done only to stimulate the cells. The stimulated HDF cells were then incubated for 48 h. Subsequently, the cell medium was collected for evaluation of MMP-1, MMP-2, MMP-3, and inflammatory cytokines.

### 2.11. Statistical Analysis

IBM SPSS Statistics for Windows, version 22 (IBM Corp., Armonk, NY, USA) was used for statistical analysis. All statistical analyses were performed using the Student’s t-test. Differences were considered statistically significant if *p* < 0.05.

## 3. Results

### 3.1. Exposure to PM10 Reduced Keratinocyte Viability and Concomitant Treatment with Saponins Improved Cell Viability

**Figure 1:**
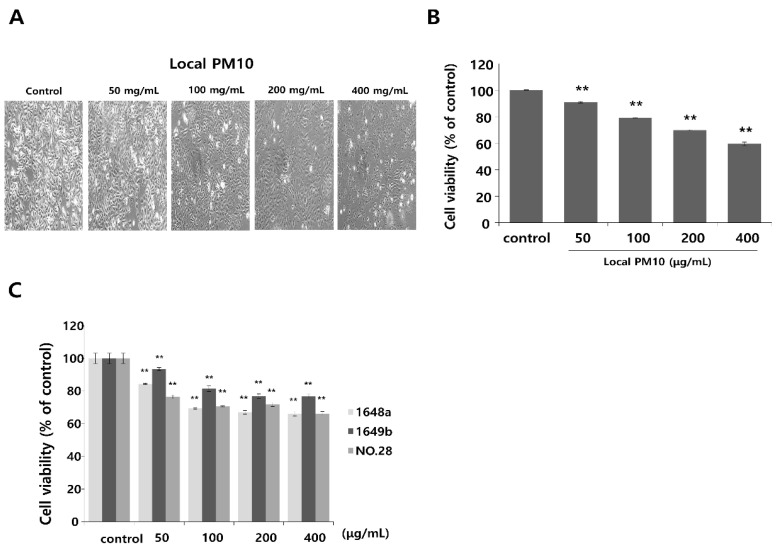
The effect of particulate matter (PM) exposure on cell viability of normal human keratinocytes (NHKs). (**A**,**B**) Significantly decreased keratinocyte cell viability was observed after treatment with different concentrations of local PM10 (original magnification: 400×). (**C**) Significantly decreased keratinocyte viability was also observed following treatment with all three different types of commercially available PM samples. ** *p* < 0.01 compared with the untreated control.

**Figure 2:**
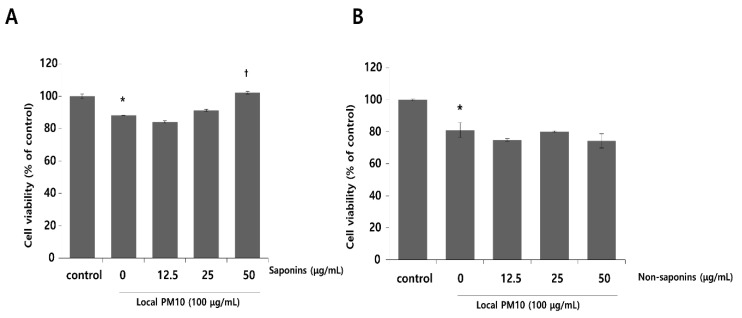
Effects of saponins and nonsaponins on PM-induced decrease in keratinocyte viability. (**A**) Concomitant treatment with 50 μg/mL saponins mitigated decreased NHK viability caused by exposure to local PM10. (**B**) On the other hand, treatment with nonsaponins did not rescue NHKs from PM-induced cytotoxicity. * *p* < 0.05 compared with the untreated control. † *p* < 0.05 compared with the PM-treated control.

Normal human epidermal keratinocytes (NHKs) were cultured with varying concentrations of local PM10 from 50 to 400 μg/mL for 24 h, and cellular viability was assessed by MTT assay. Microscopic images revealed cellular damage, with fragmentation and reduced numbers of cells after treatment with PM10 (Figure 1A). Statistical analysis demonstrated that keratinocyte viability decreased significantly and dose-dependently after exposure to four different PM10 treatments compared to the control (Figure 1B). NHKs were also treated with various commercially available PM samples, and a similar decrease in cell viability was observed at all tested concentrations (Figure 1C). Measurement of NHK viability after concomitant treatment with 100 μg/mL local PM10 and different concentrations of either saponins or nonsaponins was then performed (Figure 2). Concomitant treatments with 50 μg/mL of saponins effectively rescued NHKs from a PM10-induced decrease in cell viability, whereas concomitant treatment with nonsaponins did not promote cell viability.

### 3.2. PM10 Increased Intracellular Oxidative Stress and Concomitant Treatment with Saponins Reduced ROS Levels

**Figure 3:**
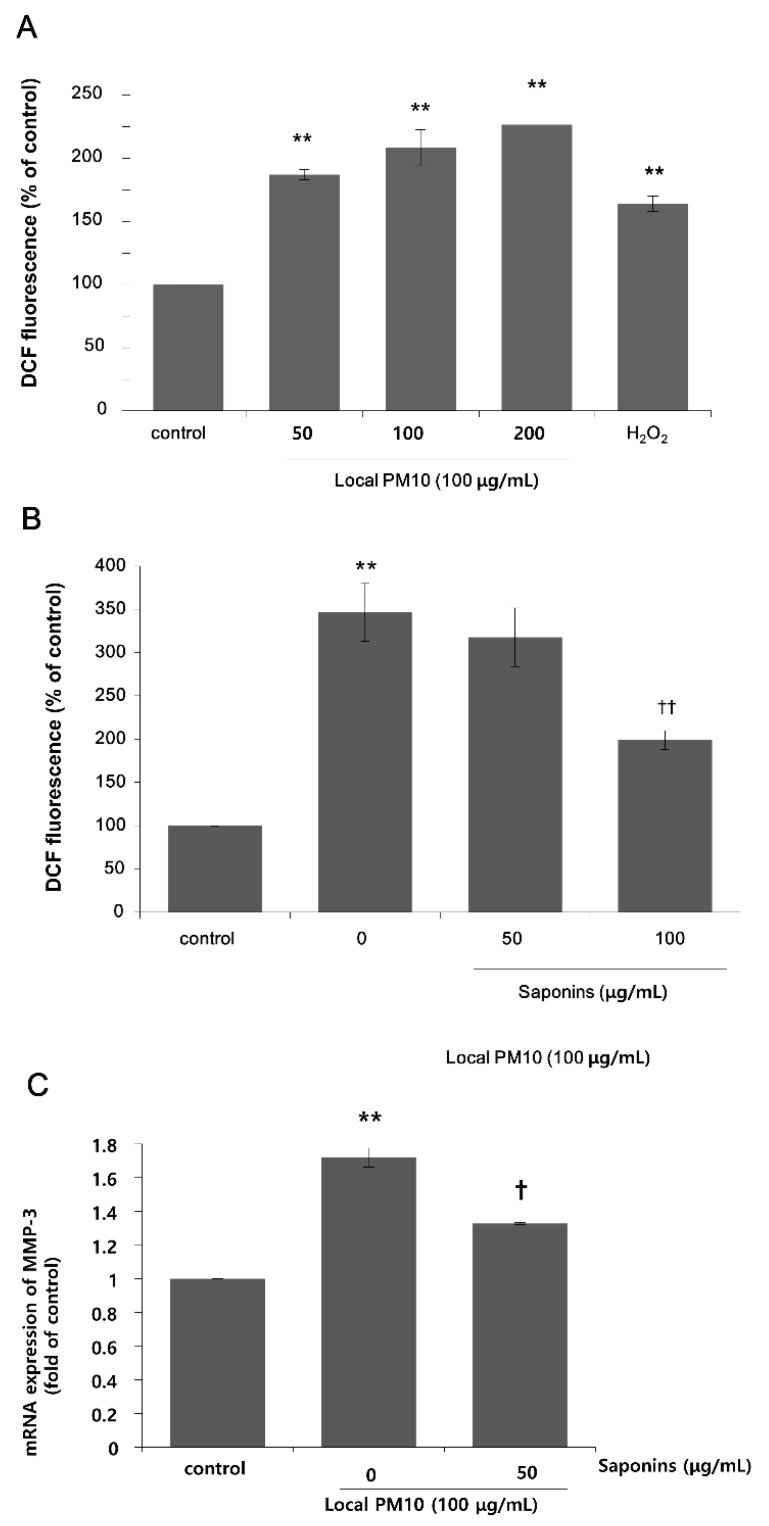
Changes in intracellular oxidative stress after exposure to local PM10, and the effects of saponins. (**A**) Exposure to local PM10 resulted in significantly increased oxidative stress in NHKs. (**B**) Treatment with 100 μg/mL saponins brought down the elevated oxidative stress caused by local PM10. (**C**) A significant increase in the mRNA expression of matrix metalloproteinase (MMP)-3 in NHKs was observed after exposure to local PM10, but this was effectively attenuated by treatment with 50 μg/mL saponins. ** *p* < 0.01 compared with the untreated control. † *p* < 0.05 and †† *p* < 0.01 compared with PM-treated control.

Next, we focused on the actions of saponins in order to investigate the possibility of using saponins against the detrimental effects of PM10, because nonsaponins did not rescue NHK viability. A DCF assay was performed using NHKs to assess intracellular oxidative stress. A dramatic increase in fluorescence levels was observed 24 h after treatment with varying concentrations of local PM10 (Figure 3A). This increase was more pronounced than that observed with H_2_O_2_ treatment, for which the ROS-producing effect was expected to be high at earlier time points. We then investigated the effect of saponins on the PM10-induced oxidative stress. The concentration of local PM10 was kept at 100 μg/mL throughout this experiment and NHKs were treated with local PM10 together with two different concentrations of saponins. Compared to the local PM10-treated control, treatment with 100 μg/mL saponins resulted in significantly decreased intracellular oxidative stress as determined by DCF assay (Figure 3B). Based on previous reports on the molecular interplay between intracellular oxidative stress and matrix metalloproteinase (MMP)-3 [23], we then evaluated the changes in the mRNA expression of MMP-3 after exposure to PM10 in NHKs, including the effect of saponins. The mRNA expression of MMP-3 increased dramatically after exposure to PM10, but this change was effectively attenuated by concomitant treatment with 50 μg/mL saponins (Figure 3C).

### 3.3. Saponins Reversed PM10-Induced Increase in the mRNA Expression of Pro-Inflammatory Cytokines

**Figure 4:**
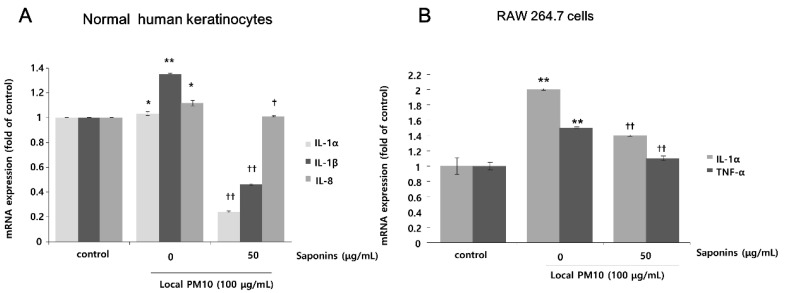
Effects of saponins on pro-inflammatory cytokine expression in human keratinocytes and RAW 264.7 cells after exposure to local PM10. (**A**) Exposure of NHKs to local PM10 caused a significant increase in the mRNA expression of interleukin (IL)-1α, IL-1β, and IL-8. However, this increase was reversed by treatment with 50 μg/mL saponins. (**B**) Similarly, significant increases in the mRNA expression of both IL-1α and TNF-α were observed when RAW 264.7 cells were treated with local PM10. However, this increase was mitigated by concomitant treatment with 50 μg/mL saponins. * *p* < 0.05 and ** *p* < 0.01 compared with the untreated control. † *p* < 0.05 and †† *p* < 0.01 compared with the PM-treated control.

The changes in mRNA expression levels of pro-inflammatory cytokines in NHKs and RAW 264.7 cells after exposure to PM10 were evaluated by qRT-PCR. Exposure of NHKs to 100 μg/mL of local PM10 led to notable increases in interleukin (IL)-1α, IL-1β, and IL-8. However, this increase in the mRNA expression level of pro-inflammatory cytokines was mitigated with concomitant treatment with 50 μg/mL saponins (Figure 4A). When RAW 264.7 cells were exposed to 100 μg/mL of local PM10, a significant increase in the mRNA expression of both IL-1α and TNF-α was observed, which was also effectively reversed by concomitant treatment with 50 μg/mL saponins.

### 3.4. PM10 Treatment Resulted in Increased mRNA Expression of Leptin, Which Was Attenuated by Treatment with Saponins

**Figure 5:**
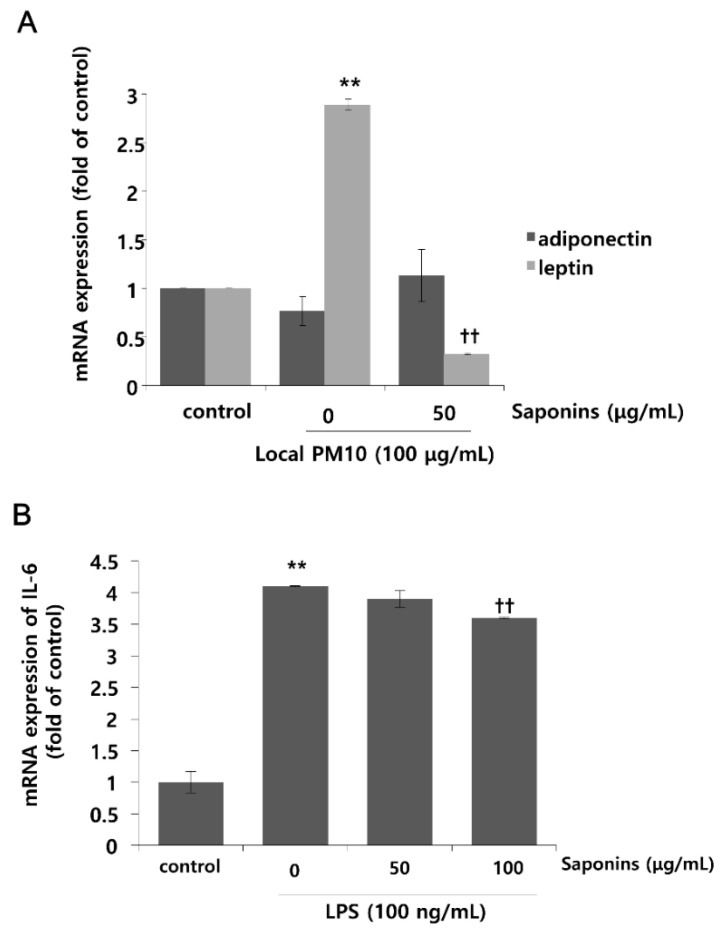
Effects of local PM10 on adipokine expressions in NHKs and the effects of saponins on adipokine expression and lipopolysaccharide (LPS)-induced inflammation. (**A**) Exposure to local PM10 significantly increased the mRNA expression level of leptin. However, this increase was effectively reversed by treatment with 50 μg/mL saponins. (**B**) A notable increase in IL-6 was observed after treatment with LPS. However, this change was significantly attenuated by treatment with 100 μg/mL saponins. ** *p* < 0.01 compared with the untreated control. †† *p* < 0.01 compared with the PM- or LPS-treated control.

Changes in the mRNA expression levels of leptin and adiponectin after exposure to local PM10 were evaluated in NHKs. Although the mRNA expression level of adiponectin was not reduced significantly after treatment with 100 μg/mL PM10, that of leptin was increased substantially (Figure 5A). Interestingly, this remarkable surge in the mRNA expression level of leptin after PM10 treatment was attenuated by concomitant treatment with 50 μg/mL saponins. Moreover, treatment with saponins mildly increased the mRNA expression of adiponectin, but this change was not found to be statistically significant. As such, in order to investigate whether saponins can mitigate the pro-inflammatory effects of adipokines, NHKs were treated with 100 ng/mL lipopolysaccharide (LPS) with or without concomitant treatment with saponins, and the mRNA expression level of IL-6 was evaluated. As expected, the expression of IL-6 was significantly increased by treatment with LPS, but this change was attenuated significantly by treatment with 100 μg/mL saponins (Figure 5B).

### 3.5. PM10 Increased Melanin Production and Saponins Attenuated PM10-Induced Melanogenesis

**Figure 6:**
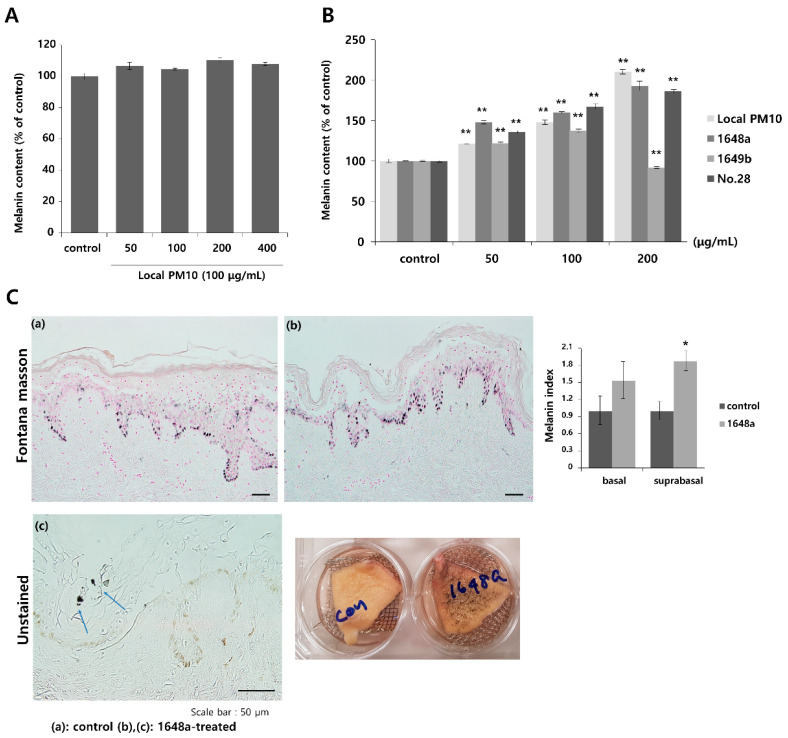
Changes in melanin content after exposure to local PM10 and the effects of saponins. (**A**) Exposure of normal human melanocytes to local PM10 did not affect the melanin content. (**B**) Treatment with each of the four PM samples, including local PM10, significantly increased the melanin content in human keratinocyte: melanocyte (1:1) coculture. (**C**) A significant increase in suprabasal melanin pigmentation was observed after treatment with 100 μg/mL 1648a on ex vivo human skin tissue (original magnification: 400×). Clumps of PM (blue arrows) can be visualized in the epidermis. * *p* < 0.01 and ** *p* < 0.01 compared with the untreated control.

**Figure 7:**
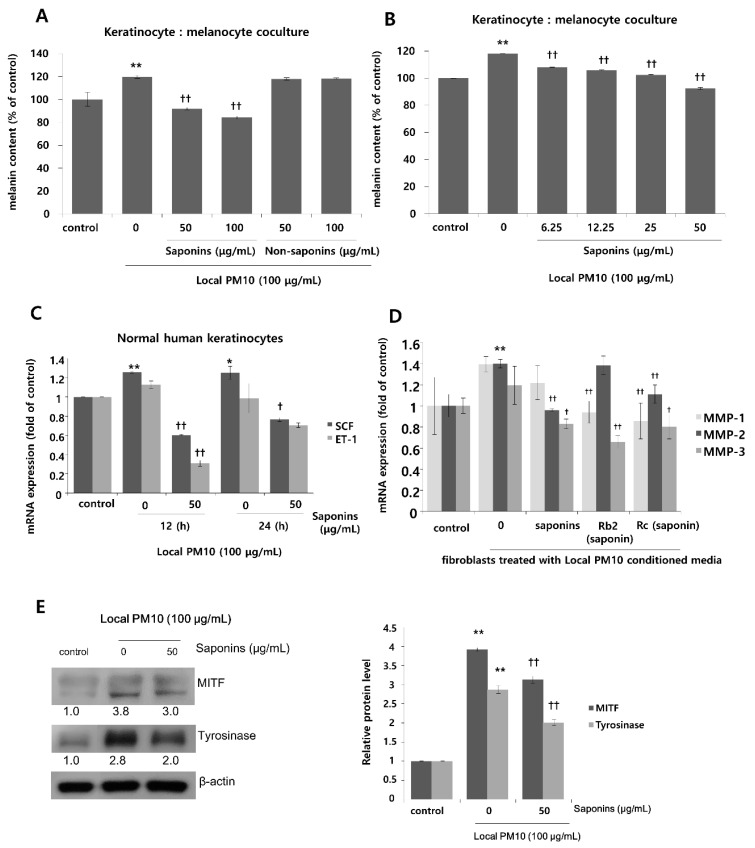
Effects of saponins on PM-induced increase in melanin content. (**A**) Treatment with saponins effectively reversed local PM10-induced increase in melanin content in keratinocyte: melanocyte (1:1) coculture, while treatment with nonsaponins did not affect melanin content. (**B**) Saponins at lower concentrations also mitigated PM-stimulated melanin production. (**C**) The mRNA expressions of both stem cell factor (SCF) and endothelin (ET)-1 were increased by treatment with PM10, while concomitant treatment with saponins decreased their mRNA expression substantially. (**D**) When human dermal fibroblasts were treated with the conditioned media obtained from keratinocytes exposed to local PM10, the mRNA expression levels of MMP-1, MMP-2, and MMP-3 were promoted. However, after treatment with various saponins, their expression levels were brought down with some variation within the effects of each tested ingredient. (**E**) Treatment with 50 μg/mL saponins downregulated expression of both MITF and tyrosinase, which were notably increased after exposure of the keratinocyte: melanocyte coculture to local PM10. * *p* < 0.05 and ** *p* < 0.01 compared with the untreated control. † *p* < 0.05 and †† *p* < 0.01 compared with PM- or conditioned media-treated control.

To investigate the effects of PM10 exposure on the production of melanin pigments, normal human melanocytes (NHMs) were treated with various concentrations of local PM10 and the melanin contents were measured on day 3. All tested concentrations of local PM10 (50, 100, 200, and 400 μg/mL) did not significantly affect the melanin content (Figure 6A). However, when the same experiment was performed with keratinocyte/melanocyte (1:1) cocultures, a significant increase in melanin content was observed at a local PM10 concentration of 50 μg/mL and above (Figure 6B). Similarly, a significant increase in melanin content was observed after treatment with three other types of PM samples. We also demonstrated that PM10 can penetrate the skin using an ex vivo skin explant model. Upon exposure to PM10, a significant increase in suprabasal melanin content was observed (Figure 6C). Keratinocyte/melanocyte cocultures were then treated with 100 μg/mL of local PM10 together with saponins at various concentrations to determine the effects of saponins on melanin production. Concomitant treatment with saponins at concentrations of both 50 and 100 μg/mL successfully attenuated the increase in melanin content by local PM10, while treatment with nonsaponins did not bring down the melanin content (Figure 7A). We additionally examined whether saponins could reduce the melanin contents at lower concentrations (from 6.25 to 50 μg/mL). Even at the lowest tested concentration of 6.25 μg/mL, concomitant treatment with saponins significantly reduced the melanin content (Figure 7B). Next, the mRNA expression of stem cell factor (SCF) and endothelin (ET)-1, both of which are well-known paracrine factors of melanogenesis secreted by keratinocytes, were measured in NHKs after treatment with 100 μg/mL of local PM10 with or without concomitant treatment with 50 μg/mL saponins. The mRNA levels of both SCF and ET-1 were found to be increased after treatment with PM10, but treatment with saponins significantly lowered their expression levels (Figure 7C). Furthermore, given that dermal MMPs are also involved in melanognesis, we evaluated how saponins affected the expression of MMPs. Human dermal fibroblasts were incubated in PM10-treated keratinocyte-conditioned media, and the mRNA expression levels of MMPs were measured. Incubation in keratinocyte-conditioned media obtained in the presence of PM10 resulted in increased levels of MMP-1, -2, and -3, but statistical significance was only observed for MMP-2 (Figure 7D). Addition of saponins, including individual saponin ingredients (Rb2 and Rc), downregulated the expression of MMPs. Finally, Western blotting was performed to evaluate the effect of saponins on the expression of melanogenesis-related genes. Treatment with 100 μg/mL of local PM10 upregulated the expression of both MITF and tyrosinase compared with the control (Figure 7E). This upregulation was reversed significantly, though not completely, by treatment with 50 μg/mL of saponins.

## 4. Discussion

The effects of air pollution on human health, and the prevention thereof, has long been a global issue. Among other pollutants, airborne particulates or particulate matter (PM) are classified as Group 1 carcinogens by the World Health Organization. Their microscopic size allows them to deeply penetrate organs and the bloodstream when inhaled. However, it was not previously clear whether PM could penetrate the skin barrier.

Recent reports have demonstrated that both PM smaller than 2.5 μm (PM2.5) and PM measuring 2.5 to 10 μm (PM10) can penetrate the stratum corneum of the skin and subsequently cause harmful reactions [21]. The results of ex vivo experiments in our study confirmed that PM10 can penetrate the skin as well as altering cutaneous physiology, including melanogenesis. In vitro investigations demonstrated that PM10 decreased keratinocyte viability at elevated concentrations. The exact mechanism by which PM10 compromises the viability of keratinocytes is uncertain; however, the dose-dependent increase in oxidative stress, as shown in this study, is suggested to be a key pathway. Other biomolecular pathways that have been indicated as being responsible for the cytotoxicity of PM include intracellular organelle dysfunction, mitochondrial damage, and autophagy [11,12,21]. The results of this study indicate the ability of saponins, a fraction of the active compounds found in Korean red ginseng (KRG), to protect keratinocytes from PM10-induced cytotoxicity by attenuating intracellular oxidative stress.

Melanin production is a strictly regulated process involving skin-resident cells, and is finely orchestrated by multiple factors including inflammatory responses as well as environmental stimuli. Although melanin pigments play an indispensable role in skin physiology by protecting the skin from ultraviolet radiation, aberrantly increased production of melanin pigments is one of the top reasons for dermatological consultations. In addition to chemicals such as fragrances, which may lead to inflammation-associated hyperpigmentation, environmental exposure to air pollutants can also result in cutaneous hyperpigmentation by provoking inflammatory reactions. Although stimulation of melanin production by PM through aryl hydrocarbon receptors (AhR) has recently been postulated, direct evidence that PM exposure leads to increased melanin synthesis was previously lacking [24]. The results of our study provide experimental evidence that exposure to PM10 promotes melanin synthesis in a dose-dependent manner. The results of Western blotting and qRT-PCR suggest that PM10 stimulates melanin production via induction of MITF and a subsequent increase in tyrosinase activity. Furthermore, changes in the inflammatory cytokine profile after PM exposure seem to play an additional role. Increased mRNA expression of pro-inflammatory cytokines including interleukin (IL)-1α, IL-1β, IL-8, and TNF-α was noted after exposure to PM10. This increase in pro-inflammatory cytokine levels was reversed by concomitant treatment with saponins. Because inflammatory reactions tend to promote melanin production, downregulation of inflammation by saponins is thought to be an important mechanism by which saponins prevent PM-induced hyperpigmentation [25,26].

Our findings indicate that suppression of leptin could be another mechanism of the anti-inflammatory and anti-melanogenetic effects of saponins. Exposure to PM not only affects the organs directly exposed, but has also been associated with systemic conditions, including alterations in glucose and lipid metabolism. Although the mechanism by which PM affects glucose and lipid metabolism has not yet been established, alteration in adipokines has been suggested as a key process [5,27]. In particular, elevated serum levels of leptin as well as leptin resistance have been characterized in studies focused on the metabolic impact of PM [8]. However, the mechanistic link between PM exposure and leptin metabolism is still unclear. Unfortunately, there are currently no effective ways to mitigate detrimental PM-induced metabolic consequences. The observation that saponin could attenuate PM-induced increase in level of leptin is thus an important finding of this study. We observed that saponins could reduce the level of leptin, which was upregulated by exposure to PM. In addition, saponins downregulated the expression of IL-6, which is a pro-inflammatory cytokine regulated by adipokines [28]. Therefore, in addition to the suppression of inflammation-associated pigmentation, our data suggest the possibility of using saponins to prevent subsequent metabolic and hormonal derangement following exposure to PM. Nevertheless, further research is needed to characterize the systemic effects of saponins in more detail.

One of the pivotal findings of this study includes the observation that KRG extracts can suppress this increase in matrix metalloproteinase (MMP) levels following PM exposure. Because MMP-induced dermal collagen fragmentation is directly affected by oxidative stress, we suspect that this phenomenon is based on the ability of KRG extracts to reduce the oxidative stress caused by PM to epidermal keratinocytes [29]. Another mechanism implicated in the suppression of MMPs by KRG extracts is the modulation of inflammatory cytokines, as observed in this study. Although regulation of MMP isoenzymes is a very complex process, pro-inflammatory cytokines including IL-1 and TNF-α are known to promote MMP production in response to various triggers [30]. Hence, we suggest that the observed decrease in MMPs by treatment with saponins involves both antioxidant and anti-inflammatory actions. In this study, a conditioned medium obtained from PM10-exposed HaCaT cells was used to simulate the dermal microenvironment after PM exposure. Theoretically, the use of human keratinocytes instead of HaCaT cells could have been ideal. However, we chose to experiment with HaCaT cells because co-cultivation of keratinocytes has several disadvantages, such as variability of cells depending on the donor, and rapid differentiation during cultivation that makes it difficult to produce high-quality conditioned media [31,32].

Certain limitations of this study need to be addressed. First, in our ex vivo skin model, PM was applied to the skin in a liquid suspension form, which is a nonstandard mode of contact. Reproducing actual environmental exposure to PM requires a well-contained chamber as well as a nebulizer, and determining the concentration or amount of PM that adheres to the skin is a challenging process. The actual physiological reactions to PM exposure could not be identified in these experiments. Second, the exact composition of local PM10 was not defined. However, we suggest that its composition should not differ greatly from that used in the study by Jin et al. [21], considering the proximity of the collection sites (16 km apart). Third, although the ex vivo skin model is expected to closely reflect cutaneous physiology, systemic factors could not be thoroughly evaluated in this study owing to the intrinsic nature of the model. Therefore, future in vivo assessment of cutaneous exposure to PM is warranted. Lastly, although treatment with saponins did not demonstrate significant cellular toxicity in our study, it has been previously reported that systemic administration of saponins in large doses can be toxic [33]. Hence, meticulous toxicological research seems necessary before the actual use of saponins against PM exposure.

In this study, significant increases in intracellular oxidative stress, expression of pro-inflammatory cytokines, leptin, and dermal MMPs were observed after treatment with PM. More importantly, melanin production was promoted by exposure to PM. These changes could all be attenuated by treatment with saponins. This study thus provides evidence that saponins possess multiphase anti-inflammatory, antioxidant, and antimelanogenetic effects on PM-exposed skin. As exposure to air pollutants including PM is virtually unavoidable, there is a need for strategies to protect the skin from the harmful effects of environmental exposure to pollutants. Future studies are thus required to determine the optimal mode of delivery of saponins, as well as effective formulations to derive clinical benefits from KRG.

## 5. Conclusions

Saponins from KRG can effectively protect the skin from the detrimental consequences of exposure to PM10. Saponins reduced PM-induced intracellular oxidative stress, which improved keratinocyte viability and prevented adipokine-associated inflammation and pigmentation. Elevated levels of leptin were mitigated by treatment with saponins. In addition, saponins successfully reduced excessive melanin accumulation caused by PM exposure. Therefore, saponins could be a potential agent that can be used to treat PM-induced cutaneous changes including inflammation and hyperpigmentation.

## Data Availability

All data is contained within the article.
